# Supplementary material for: The interactome of intact mitochondria by cross-linking mass spectrometry provides evidence for coexisting respiratory supercomplexes
Source: Mol Cell Proteomics. 2017 Dec 8;17(2):216–32. doi: 10.1074/mcp.RA117.000470 (PMC5795388; doi:10.1074/mcp.RA117.000470)
Supplement: Supplemental Data [file supp_RA117.000470_133922_0_supp_23978_fzffwf.zip › spectra_annotation/mito_spectra_annotation/70-2-1-1-4-1.pdf]

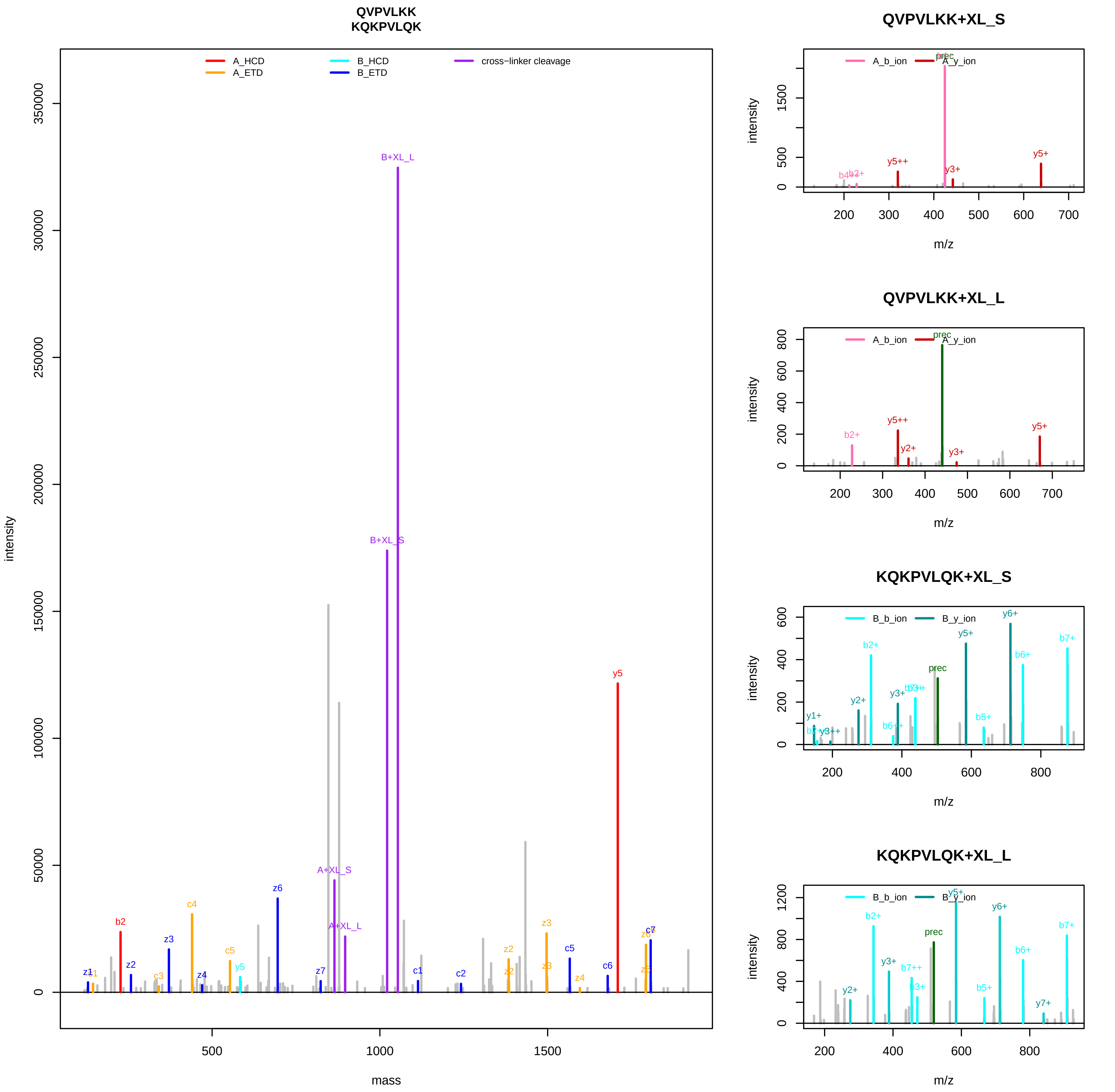
QVPVLKK
KQKPVLQK
A_HCD
A_ETD
B_HCD
B_ETD
cross−linker cleavage
intensity
mass
B+XL_L
B+XL_S
A+XL_S
A+XL_L
y5
z6
c4
z3
z2
c5
b2
y5
z1
c3
z4
z7
c1
c2
z3
c5
c6
z4
c7
z6
QVPVLKK+XL_S
A_b_ion
A_y_ion
prec
y5++
y3+
y5+
b4++
b2+
m/z
QVPVLKK+XL_L
b2+
y2+
y3+
KQKPVLQK+XL_S
B_b_ion
B_y_ion
y6+
y5+
b7+
b6+
b2+
y2+
y3+
b3+
b5+
b6++
y1+
y3++
KQKPVLQK+XL_L
b7++
y7+
